# Point-of-care brain injury evaluation of conscious awareness: wide scale deployment of portable HCS EEG evaluation

**DOI:** 10.1093/nc/niy011

**Published:** 2018-11-23

**Authors:** Carolyn M Fleck-Prediger, Sujoy Ghosh Hajra, Careesa C Liu, D Shaun Gray, Donald F Weaver, Shishir Gopinath, Bruce D Dick, Ryan C N D’Arcy

**Affiliations:** 1 Neuroscience and Mental Health Institute, University of Alberta, Canada; 2 Halvar Jonson Centre for Brain Injury, Canada; 3 Faculty of Applied Sciences (Engineering Science and Computing Science), Simon Fraser University, Canada; 4 NeuroTech Lab, Simon Fraser University and Fraser Health Authority, Surrey, BC, Canada; 5 Division of Physical Medicine and Rehabilitation, University of Alberta, Canada; 6 Krembil Research Institute, UHN, University of Toronto, Canada; 7 Departments of Anesthesiology and Pain Medicine, Psychiatry & Pediatrics, University of Alberta, Canada

**Keywords:** Disorders of Consciousness, EEG, ERP, Halifax Consciousness Scanner, P300

## Abstract

Survivors of severe brain injury may remain in a decreased state of conscious awareness for an extended period of time. Clinical scales are used to describe levels of consciousness but rely on behavioural responses, precipitating misdiagnosis. We have previously utilized event-related potentials (ERPs) to circumvent reliance on behavioural responses. However, practical implementation barriers limit the clinical utility of ERP assessment at point-of-care (POC). To address this challenge, we developed the Halifax Consciousness Scanner (HCS)—a rapid, semi-automated electroencephalography system. The current study evaluated: (i) HCS feasibility in sub-acute, POC settings nationwide; (ii) ERP P300 responses in patients with acquired brain injury versus healthy controls; and (iii) correlations within and between clinical measures and P300 latencies. We assessed 28 patients with severe, chronic impairments from brain injuries and contrasted the results with healthy control data (*n* = 100). Correlational analyses examined relationships between P300 latencies and the commonly used clinical scales. P300 latencies were significantly delayed in patients compared to healthy controls (*P* < 0.05). Clinical assessment scores were significantly inter-correlated and correlated significantly with P300 latencies (*P* < 0.05). In sub-acute and chronic care settings, the HCS provided a physiological measure of neurocognitive processing at POC for patients with severe acquired brain injury, including those with disorders of consciousness.

## Introduction

After serious neurological injury, patients may die, remain in coma or awaken as evidenced by eye opening. Those who awaken may remain in a state of environmentally unresponsive wakefulness, improve to a minimally conscious state (MCS) with clear but intermittent and inconsistent signs of self and environmental awareness or regain full conscious awareness (Di Perri *et al.* 2014). Edlow *et al.* (2017) suggest that early detection of masked consciousness and cortical responses could inform life-altering clinical decision-making. However, medical complications and the related interventions often impede accurate evaluation of consciousness (Giacino *et al.* 2013). Given these confounds, assessing a patient’s level of consciousness (LOC) too early may misinform clinical decision-making at the top of the critical care cascade. During acute phases, many patients may truly be incapable of functional information processing but in some cases, consciousness gradually recovers. This cognitive recovery can happen with or without the development of motor capacities and behavioural output. In view of this and the fact that subtle changes can go unnoticed in busy long-term care settings, Giacino *et al.* (2014) stress the importance of an integrated system of care that responds to the needs of patients as they evolve.

Clinical assessments such as the Glasgow Coma Scale (GCS) (Teasdale and Jennett 1974) and Coma Recovery Scale-R (CRS-R) (Giacino *et al.* 2004) rely on the subjective observation of patient responses without considering patient or situational variables (Reith *et al.* 2016). Scales that are solely based on observation of patient responses misdiagnose certain patients because consciousness can exist without behavioural signs. In fact, as Wijnen *et al.* (2007) point out, patients who remain in an unresponsive wakeful state do not score worse on early motor-based assessment scales than those who eventually regain some degree of conscious awareness. Andrews *et al.* (1996) examined patients on a rehabilitation unit with the working diagnosis of vegetative state and found the misdiagnosis rate to be as high as 43%. Importantly, once conscious awareness was detected, nearly all of these patients were able to relay choices regarding quality of life issues using alternate means of communication. Despite the growing recognition of this important problem, Schnakers *et al.* (2009) showed that the rate of misdiagnosis did not change substantially over the 15-year period following the study by Andrews *et al.* (1996), remaining at over 40%. This situation underscores the need for objective physiological measurement tools that bridge the gap between research evidence and clinical implementation. Solutions are emerging from brain imaging technologies that track physiological responses and these tactics are being translated to sub-acute rehabilitation settings. Fleck-Prediger *et al.* (2015) used the HCS in a traumatic brain injury (TBI) case study to evaluate event-related potential (ERP) changes during active speech language rehabilitation. In this single case study, P300 results remained stable while the response size of a later ERP component, the N400, improved in parallel with significant clinical gains in auditory comprehension.

A number of groups, including ours, have used brain imaging technologies such as electroencephalography (EEG)/ERPs, positron emission tomography (PET) and functional magnetic resonance imaging (fMRI) to explore Disorders of Consciousness (DoC) (Laureys *et al.* 2004; Owen *et al.* 2006; Gawryluk *et al.* 2010; Casali *et al.* 2013; Harrison and Connolly 2013; Ragazzoni *et al.* 2013; Sitt *et al.* 2014; Bodart *et al.* 2015; Sabino *et al.* 2017). While these various brain-imaging technologies have contributed valuable insights, one of the major practical challenges has been clinical implementation in front-line point-of-care (POC) settings. To address this, we developed a portable, semi-automated EEG device, the Halifax Consciousness Scanner (HCS), for user-friendly ERP testing (D'Arcy *et al.* 2011). The HCS provides an objective, rapid POC approach and has been separately validated across a large sample of healthy controls (Sculthorpe-Petley *et al.* 2015).

With advances in portable EEG devices, ERPs are increasingly being used to investigate conscious awareness (Hinterberger *et al.* 2005; Cruse *et al.* 2011; D'Arcy *et al.* 2011; Fleck-Prediger *et al.* 2015; Guger *et al.* 2017). Emerging from clinical ERP assessment work that began the mid-1990s (Connolly *et al.* 1995, 1999, 2000; Connolly and D’Arcy, 2000; D'Arcy *et al.* 2003; Hajra *et al.* 2016), the objective of the HCS was to integrate a range of ERP components into a rapid, semi-automated evaluation for POC. Any one or more of these ERP components could then be utilized for neuroscience evaluations from low-level sensation to higher-level language and cognition. With the HCS normative study complete (Sculthorpe-Petley *et al.* 2015) and preliminary case study evidence (Fleck-Prediger *et al.* 2015), patient studies that evaluate the practical applications of this ERP assessment across different DoC POC sites are underway to further develop and validate the technology. In this study, the compressed HCS enabled evaluation of the relationship between auditory evoked P300 responses and subjective clinical DoC measures (i.e. rating scales). In order to ensure scientific rigor and avoid spurious conclusions, we purposefully targeted a single robust measure appropriate for complex patient data (i.e. P300 latency). We systematically required the presence of the N100 to validate auditory sensation and then tested the null hypothesis that the P300 latency (as a neural measure of information processing) would not show a significant relationship with the clinical rating scales.

The P300, an objective, physiological measure of information processing, is a positive endogenous component with a prototypical peak 300 ms after stimulus onset, usually between 250 and 500 ms but this range can vary with the stimulus modality (Polich 2007). It is thought to serve as a temporal measure of the neural activity underlying the allocation of attention and immediate memory processes (Polich and Heine 1996). In simple tasks, the P300 amplitude is typically large and its latency is short in duration. However, as task demands increase, the amplitude decreases and the peak latency lengthens because processing resources must be dedicated to task completion (Kok, 2001). Therefore, we anticipate the P300 latency will be delayed in DOC patients (relative to normative data) and will correlate negatively with the patients’ measured state of conscious awareness using standard clinical tests. As Steppacher *et al.* (2013) have shown, this does not to imply that P300 has significant predictive powers regarding the re-emergence of consciousness. Rather, the intent is to establish the HCS evoked auditory P300 as a neural indicator of information processing in patients with lower levels of conscious awareness.

Research and rehabilitation communities often do not adequately monitor patients over time and therefore may not detect subtle changes in conscious awareness. The need for serial monitoring is based on an emerging understanding that patients can demonstrate substantial recovery over long periods of time. For example, Nakase-Richardson *et al.* (2012) studied acute and long-term outcomes from DoC and found that two-thirds of patients regained the ability to follow commands during rehabilitation and one-fourth emerged from post-traumatic amnesia. Furthermore, significant recovery continued for 2 years post-injury with more modest gains for as long as 5 years post-injury. We have also reported on a 3-year case control study in which a severe TBI survivor recovered from coma. He demonstrated recovery of motor function and corresponding fMRI activation changes more than 6 years after injury (D'Arcy *et al.* 2016).

## Objectives and Hypothesis

We conducted a validation study to test the HCS in clinical, sub-acute acquired brain injury settings nationwide. The objectives were to (i) evaluate the feasibility of HCS testing at POC centres nationwide; (ii) compare the P300 response generated by HCS to normative data; and (iii) examine the correlations within and between clinical scales to P300 latencies. It was hypothesized that: (i) patient P300 latencies would be delayed relative to healthy control normative data; (ii) that the GCS, CRS-R and Functional Independence Measure (FIM) (Keith *et al.* 1987) clinical scales would be significantly inter-correlated; and (iii) patient P300 latencies would also be significantly correlated with the above clinical scales, demonstrating an important relationship with functional impairment.

## Methods

### Participants

**Figure 1.**
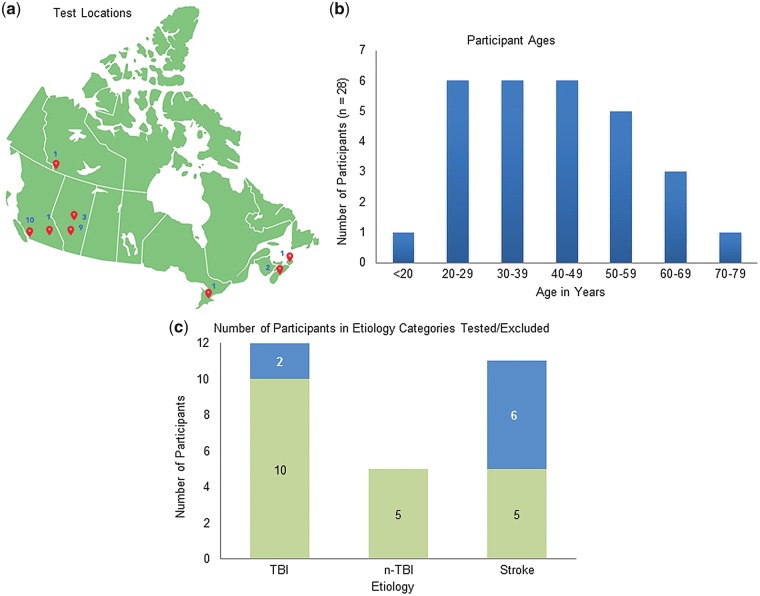
(**a**) Locations 28 patients were tested across Canada. (**b**) Number of patients (y-axis) tested in each age range (x-axis) in years. (**c**) Number of patients (y-axis) tested in each aetiology category—TBI, n-TBI and stroke (x-axis) with green (bottom) showing number of patients successfully tested and blue (top) showing number of patients tested but excluded.

EEG testing was attempted on 28 adults with severe neurological injury at diverse points-of-care across Canada (Fig. 1a). Caregivers or therapists referred patients, and preferentially included those patients suspected to have some degree of awareness. The EEG quality was sufficient to evaluate HCS results in 20 of the cases. HCS results from the remaining eight participants were not analysed due to: hearing impairment (*n* = 1), poor signal quality/extreme environmental and/or muscle movement artefact (*n* = 5) or technical failure (*n* = 2) (Table 1). All participants had sustained severe acquired brain injury (traumatic or non-traumatic including anoxia) or stroke (haemorrhagic or ischaemic) with a GCS of 8 or less in the acute phase (Table 2). Participants were medically stable but chronically impaired. There was heterogeneity in terms of age (Table 1 and Fig. 1b), aetiology (Table 1 and Fig. 1c), time elapsed since injury or event (Table 1), level of responsiveness (Table 3) and rehabilitation opportunity. Twenty-five percent of these participants were fully conscious but experienced persistent and severe motor, communication and cognitive sequelae consequent to their neurological injury. The remaining 75% of the participants were classified as either being comatose, unresponsive but wakeful, partially/inconsistently responsive or fully responsive based on clinical observations and the administration of the JFK CRS-R. Although our clinical categories were informed by using the CRS-R, we purposefully avoided categorizing the patients into the firm unresponsive wakeful syndrome (UWS) or minimally conscious state (MCS) divisions described by the CRS-R, as the goal was to differentiate between broad levels of responsiveness using an objective, physiological measure not to assign patients to specific diagnostic categories.

**Table 1:** Demographics including sex, age, aetiology of injury and time post injury for 20 patients successfully tested (top). Patients tested but excluded from results with the reason for exclusion specified (bottom)

#	Sex	Age	Aetiology	Time post	#	Sex	Age	Aetiology	Time post
1	M	43	TBI	38 m	2	M	22	TBI	15 m
3	M	26	n-TBI	44 m	4	F	67	Stroke	35 m
5	M	57	TBI	98 m	6	M	30	n-TBI	31 m
7	M	34	n-TBI	199 m	8	F	64	Stroke	214 m
9	M	45	TBI	7 m	10	F	43	TBI	19 m
11	M	55	Stroke	20 m	12	F	54	Stroke	20 m
13	M	27	TBI	12 m	14	M	46	TBI	62 m
15	M	35	TBI	54 m	16	F	24	TBI	11 m
17	M	18	TBI	27 m	18	M	57	Stroke	6 d
19	F	36	n-TBI	130 m	20	M	71	n-TBI	11d

Exclusions									

#	Behavioural diagnosis			Exclusion reason	#	Behavioural diagnosis			Exclusion reason

21	Conscious			Environmental artefact	22	Conscious			Environmental artefact
23	Conscious				24	Conscious			
25	Conscious			Cranioplasty	26	Conscious			Poor hearing
27	Conscious			ERP trigger issues	28	Conscious			ERP trigger issues

Demographics of participants and rationale for exclusion.

**Table 2:** Range, mean, and standard deviation for time of onset to testing, CRS-R (at testing) and GCS (at injury and testing) for participants with TBI, n-TBI, stroke and combined sample

Aetiology	*n*	Time to testing **(Range),**$\;\bar{x}$**, σ**	CRS-R **[Range],**$\;\bar{x}$**, σ**	GCS at injury **[Range],**$\;\bar{x}$**, σ**	GCS at testing **[Range],**$\;\bar{x}$**, σ**
TBI	10	[7 months–8.2 years] $\bar{x\;}$= 2.9 years σ = 2.4 years	[6–22] $\bar{x\;}$= 13.4 σ = 5.2	[3T–7T] $\bar{x\;}$= 3.9 σ = 1.4	[7T–15] $\bar{x\;}$= 9.4 σ = 2.5
n-TBI	5	[11 days–16.6 years] $\bar{x\;}$= 6.7 years σ = 6.8 years	[1–17] $\bar{x\;}$= 9.8 σ = 6.7	[3T–5T] $\bar{x\;}$= 3.4 σ = 0.9	[5T–12] $\bar{x\;}$= 9 σ = 2.9
Stroke	5	[6 days–17.8 years] $\bar{x\;}$= 4.8 years σ = 7.3 years	[11-18] $\bar{x\;}$= 12.8 σ = 3.0	[3T-7T] $\bar{x\;}$= 3.8 σ = 1.8	[7T-11] $\bar{x\;}$= 9 σ = 1.6
Combined	20	[6 days–17.8 years] $\bar{x\;}$ = 4.3 years σ = 5.2 years	[1–22] $\bar{x\;}$= 12.4 σ = 5.2	[3T–7T] $\bar{x\;}$= 3.8 σ = 1.3	[5T–15] $\bar{x\;}$= 9.2 σ = 2.3

Time to testing information and clinical scores (CRS-R and GCS).

**Table 3:** Participant specific clinical assessment scales: GCS and CRS-R at time of testing with sum in brackets

#	GCS	CRS-R	Clinical impression	#	GCS	CRS-R	Clinical impression
**1**	15	4, 5, 6, 2, 2, 3 (22)	Responsive	11	8T	2, 3, 3, 1, 0, 2 (11)	Partially responsive
**2**	12	4, 5, 6, 2, 2, 3 (22)	Responsive	12	9	2, 3, 3, 1, 0, 2 (11)	Partially responsive
**3**	12	3, 5, 5, 1, 1, 2 (17)	Responsive	13	8	2, 3, 3, 1, 0, 2 (11)	Partially responsive
**4**	11	3, 5, 5, 2, 1, 2 (18)	Responsive	14	7T	2, 3, 3, 1, 0, 2 (11)	Partially responsive
**5**	11	3, 5, 3, 1, 2, 2 (16)	Responsive	15	8	2, 3, 3, 1, 0, 2 (11)	Partially responsive
**6**	11	2, 3, 3, 1, 0, 2 (11)	Partially responsive	16	8	2, 2, 3, 1, 0, 2 (10)	Partially responsive
**7**	10	2, 3, 5, 2, 1, 2 (15)	Partially responsive	17	8	1, 1, 2, 1, 0, 1 (6)	Unresponsive
**8**	10	2, 3, 5, 1, 0, 2 (13)	Partially responsive	18	7T	1, 1, 1, 1, 0, 1 (5)	Unresponsive
**9**	9	2, 3, 5, 2, 0, 2 (14)	Partially responsive	19	7	1, 1, 1, 1, 0, 1 (5)	Unresponsive
10	8	2, 3, 3, 1, 0, 2 (11)	Partially responsive	20	5T	0, 0, 1, 0, 0, 0 (1)	Comatose

Clinical scores and clinical impression of responsiveness.

Except for the comatose patient, all patients who were successfully tested awoke to, startled at, or oriented towards out of sight noise—a behavioural indicator of intact hearing. Three participants received full audiology evaluation to ensure their capacity to participate.

### Clinical scale scores

The GCS and JFK CRS-R scores were collected as clinical measures at the time of testing. All clinical measures were correlated with one another and with P300 latencies. P300 latencies were also correlated with the FIM on a subset of participants engaged in inter-disciplinary rehabilitation. FIM scores were included in this study as this tool is frequently used in clinical settings—even for patients who are not fully responsive. In addition, this sample included a wide variety of patients with severe brain injury, including those who were unresponsive, minimally responsive and fully responsive, and the FIM captured functional differences between the groups. As the FIM reflected, many survivors of severe brain injury regain full consciousness and compensate well for their impairments despite persistent physical impairments.

### Instrumentation

HCS used a portable 8-channel GmobiLab EEG system (g.tec Medical Engineering, GmbH), comprised of recording electrodes, earphones, an electrode interface, an impedance monitor and a handheld computer. Custom software automated auditory stimulus presentation (5-min sequence) and data acquisition, with a semi-automated data analysis that was manually verified. Results were derived from three midline recording electrodes, covering the anterior–posterior axis (approximating Fz, Cz, Pz). Four other electrodes served as ground (forehead), reference (earlobe) and left and right electro-oculograms (EOG) (Connolly and Kleinman 1978). All impedances were below 5 kΩ. The EEG and EOG signals were sampled at a rate of 256 Hz, with a band-pass of 0.1–100 Hz and stored for offline analyses.

### Process

The National Research Council of Canada (NRC) and University of Alberta Human Research Ethics Board approved the study. Each patient or a legal delegate provided informed consent. During a single visit, the examiner(s) administered the HCS paradigm twice and administered clinical consciousness scales (GCS-R and CRS-R), often recruiting assistance from the rehabilitation staff and/or nursing staff familiar with the patient. An inter-disciplinary group of rehabilitation professionals collaboratively ranked the subset of patients actively participating in rehabilitation on the FIM as part of routine clinical care.

ERP analyses were completed and P300 components were both automatically identified and manually verified (SGH, CCL and RCND). All P300 component identification results were then additionally evaluated by separate examiners blinded to patient identities and profiles (CfP and BD). Patient preparation for HCS testing involved simple instructions to listen to tones and sentences for anything unexpected.

### Stimuli

Details of the HCS stimulus sequence have been described elsewhere (D'Arcy *et al.* 2011). Briefly, the HCS elicited auditory ERP components linked to sensation (N100); perception (MMN); attention (P300), memory for own name (Early Negative Enhancement to Sound of Own Name); and comprehension (N400). The 5-minute auditory stimulus sequence was comprised of tones (2.5 min) followed by speech (2.5 min). Amplitude and latency data were collected on all components. For the purpose of a comparison across clinical tests, the current study focused specifically on presence or absence of N100 (sensation) and P300 latency, a well-established ERP measure of information processing. Other ERP component measures are being analysed, and the results will be detailed in future publications. In the present study, after screening for an N100 response, patient P300 latencies were compared to those of 100 healthy normative controls and then correlated with patients’ clinical scores on the GCS, CRS-R and FIM (selected cases).

### Data analysis

In order to address challenges related to POC clinical testing in severe brain injury, the EEG analysis involved advanced methods to ensure proper identification of the P300 latency. Data analysis was performed using a combination of BrainVision Analyzer 2 (Brain Products GmbH, Germany) and custom software in MATLAB (MathWorks, USA). Raw continuous EEG data were band pass filtered to 0.1–20 Hz, then visually inspected to reject segments containing artefacts. Temporal independent component analysis (ICA) was performed for blink detection, followed by ocular correction using the Gratton and Coles method (Gratton *et al.* 1983) Subsequent pre-processing was carried out according to established methods (Luck 2014), comprised of band pass filtering (0.1–10 Hz), segmentation (−100 to 900 ms) of epochs, baseline correction (−100 to 0 ms) and conditional averaging. To further enhance the signal-to-noise ratio and optimize component detection for a heterogeneous patient sample from multiple clinical sites; segmented data were processed using wavelet filtering prior to trial averaging to obtain ERPs (Daubechies 1990). In usual ERP practice, across-trial averaging is employed to enhance the signal-to-noise ratio in order to isolate event-related brain potentials that are often several magnitudes smaller than background EEG. However, these often require a large number of trials, which is impractical within a clinical setting where signal-to-noise is suboptimal. Accordingly, we utilized a recently developed alternate SNR enhancing approach using wavelet filtering. The wavelet method is well-suited to non-stationary ERP analysis (Demiralp *et al.* 1999). The wavelet filtering technique builds upon previous literature (Hu *et al.* 2010) and uses the sample of 100 healthy control ERP data to derive thresholds for filtering patient data. Specifically, continuous wavelet transform (CWT) was first applied to the grand-average ERP waveform of the healthy control data as follows:

$$X\left(t,f\right)=\;\int_{\tau }x\left(t\right)\cdot \;\sqrt{\frac{f}{f_{0}}}\;\cdot \;\psi^{*}\;\left(\frac{f}{f_{0}}\;\cdot (\tau -t)\right)\;d\tau$$

$$\psi \left(t\right)=\;\frac{1}{\sqrt{\Pi }f_{b}}\;e^{2i\pi f_{0}x}\;e^{\frac{-x^{2}}{f_{b}}}$$

where $x\left(t\right)$ is the original grand-average ERP signal in time domain, $X\left(t,f\right)$ is the transformed signal in time-frequency domain, $\psi \left(t\right)$ is the mother wavelet in the form of a complex Morlet function with central frequency $f_{0}$, and t and f are the time and frequency indices, respectively. The parameters $f_{b}$ and $f_{0}$ were set to 0.05 and 6, respectively, in accordance with previous literature (Hu *et al.* 2010).

The power spectrum was computed from the transformed time-frequency signal as the square of the magnitude of the wavelet coefficients and was baseline corrected by subtracting the mean of the spectral power during the 100 ms pre-stimulus interval. The power spectrum was then normalized, and the cumulative distribution function (CDF) computed. The dynamic range of the CDF was thereafter derived, and the filtering threshold was determined as the wavelet coefficient corresponding to 85th percentile of the CDF. Subsequently CWT was computed for single trial data for each patient, and all resulting wavelet coefficients below the threshold level were set to zero. The filtered trial-level spectra were then converted back to time domain via inverse continuous wavelet transform (iCWT) as below:

$$y\left(t\right)=\;C_{\psi }\;\int_{\tau }^{\;}\int_{f}^{\;}X^{'}\left(t,f\right)\cdot \;\sqrt{\frac{f}{f_{0}}}\;\cdot \;\psi (\frac{f}{f_{0}}(t-\;\tau ))\cdot {\left(\frac{f}{f_{0}}\right)}^{2}\;d\tau \;df\;$$

where $y\left(t\right)$ is the converted signal in time domain, $X^{'}\left(t,f\right)$ is the wavelet transform after filtering, $C_{\psi }$ is a scalar normalization coefficient and other quantities are defined as previously illustrated. Finally, the wavelet-filtered trial-level data for each patient were averaged to derive the ERP waveform for that patient. Although CWT preserves non-phase-locked information, the application of iCWT prior to conditional trial-averaging for ERP generation ensures that the final waveforms contain only responses that are both time- and phase-locked, in line with traditional ERP practice (Luck 2014).

Both healthy control and patient data were subjected to wavelet filtering prior to ERP derivation and the same filtering thresholds were used in both healthy control and patient data. The results reported herein thus focused on relative differences between the two groups rather than raw numerical values. The current study focused on evaluation of P300 latencies only and did not compare P300 amplitudes between the healthy and patient groups because the wavelet filtering method is known to reduce ERP amplitudes but does not significantly impact component latencies (Hu *et al.* 2010). Statistical significance was evaluated between the control and patient groups using Welch’s *t*-test. In order to determine whether differences in outcomes existed between TBI and non-traumatic brain injury (n-TBI) populations, the TBI sub-group was also compared to other patients and healthy controls (corrected for multiple comparisons).

Further statistical analyses were performed to evaluate the effect of unequal subject numbers between the control and patient groups. This involved randomly selecting a sub-group of healthy control participants equal in number to that of the patient group, and repeating the Welch’s *t*-test. This process was repeated 10 000 times following randomized sub-group selections, and the mean probabilities were computed.

Patient ERP results were excluded if there was no clear N100 response to the tones. Two separate groups of blinded examiners (RCND, SGH, CL versus BD and CfP) reviewed the data to identify presence or absence of the N100 and P300 responses. Inter-rater reliability for the P300 was 95% (19/20) for averaged responses. However, when the individual (versus averaged) waveforms were evaluated for the patient (Participant 19), concordance was reached.

## Results

**Figure 2.**
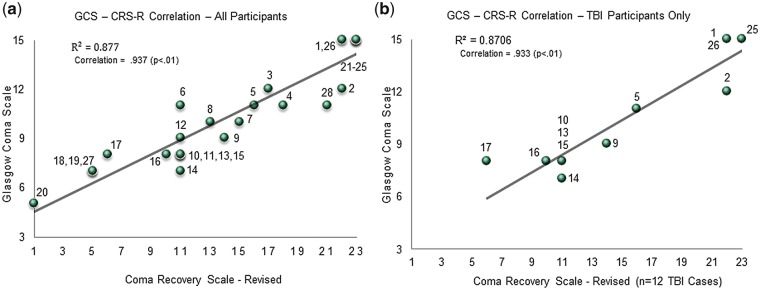
(**a**) GCS (y-axis) versus CRS-R score (x-axis) correlation with participant numbers labelled for all 28 patients. (**b**) GCS (y-axis) versus CRS-R score (x-axis) correlation with participant numbers labelled for 12 TBI patients.

**Figure 3.**
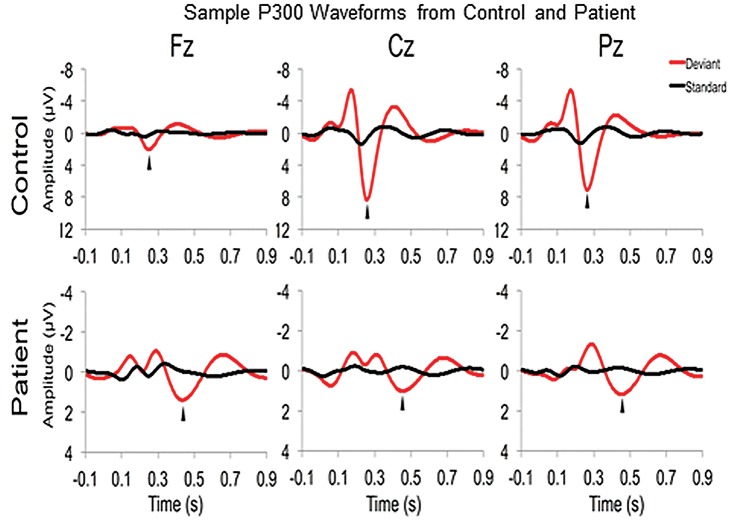
Sample P300 wavelet based waveforms from a healthy control and a representative patient participant.

**Figure 4.**
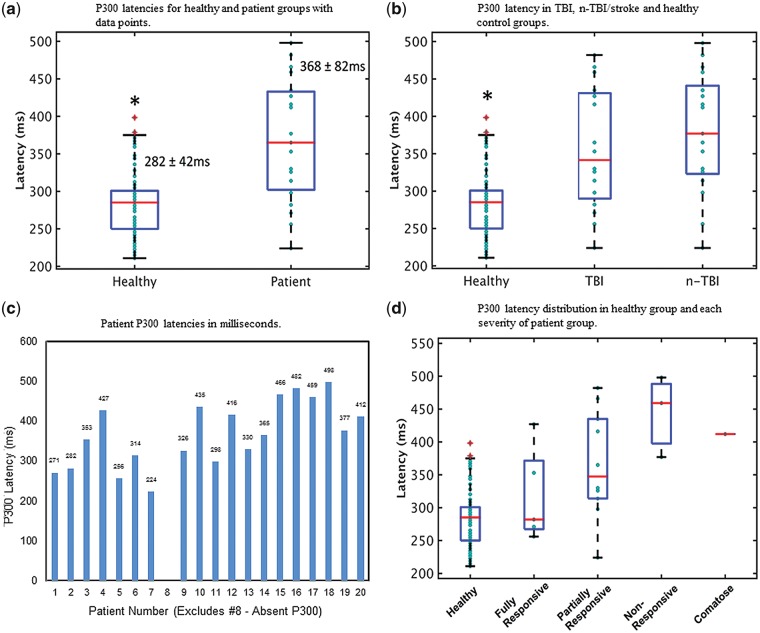
(**a**) Box plots showing P300 latencies for the healthy (*N* = 100) and patient (*N* = 19) groups, with individual data points overlaid. Blue boxes denote 25th–75th percentile ranges, while the red bars indicate median in each group. Numbers are also displayed corresponding to the mean ± SD of each group. *P < 0.05. (**b**) Box plots showing P300 latencies for TBI (*N* = 10), non-TBI (*N* = 9) and healthy (*N* = 100) groups. For each group blue boxes denote quartile range, red bar indicates median and red crosses represent data points that are more than 1.5 times away from the nearest quartile boundary. Cyan circles correspond to individual data points. *P < 0.05 compared to other groups. (**c**) P300 latency in milliseconds (y-axis) for each participant (numbered on x-axis, *n* = 19 as participant 8 did not demonstrate a P300 response). (**d**) Distribution of P300 latencies in each group, including healthy (*N* = 100), fully conscious (*N* = 5), partially conscious (*N* = 10), non-responsive (*N* = 3) and comatose (*N* = 1). For each clinical severity the blue boxes denote quartile range, red line represents median and red crosses indicate outliers more than 1.5 times away from the nearest quartile boundary. Cyan circles correspond to individual data points.

Figure 2a shows the correlation between the GCS and CRS-R scores for all 28 patients (*r* = 0.937, *P* < 0.01). Figure 2b demonstrates the correlation (0.933) is significant (*P* < 0.01) specifically for the TBI only group (*n* = 12). Figure 3 shows a representative P300 response for a healthy control and a patient participant. In keeping with previous work (Picton 1992; Perrin *et al.* 2005; Linden 2005), patients in this study with severe stroke and brain injury (*n* = 20) showed delayed P300 latencies compared to those of the 100 healthy controls (*P* < 0.05) (Sculthorpe-Petley *et al.* 2015). Participant 8, who was partially/inconsistently responsive, did not show a P300 response so the averages were based on 19 individuals. The mean P300 latency for patients (*n* = 19) was 368 ms (SD = 82 ms), whereas the mean P300 latency for 100 healthy controls was 282 ms (SD = 42 ms). This result was not affected by the unequal subject numbers between the two groups, as statistical significance was maintained even when an equal number of control participants were randomly selected from the 100 overall control population and compared to patients (*P* < 0.05). Bootstrapping confidence interval (CI) analysis was performed by randomly selecting a subsample of 10 participants from each of the healthy and patient groups, and computing the group means from the subsample. Significance remained when this was repeated 10 000 times. The graphic corresponding to the permutation statistic is included as Supplementary Fig. SM1. Figure 4a is a box plot showing the mean P300 latencies (±SD) for the healthy (*N* = 100) and patient (*N* = 19) groups, with individual data points overlaid.

As shown in Fig. 4b, the group means were 282.1 ± 41.6 ms for healthy controls, 367.2 ± 86.9 ms for TBI and 368.8 ± 81.8 ms for n-TBI including stroke. The TBI and n-TBI/stroke groups’ mean P300 latencies were not significantly different from each other. All groups were compared to each other with two tailed 2-sample unequal variance *t*-tests, with the TBI and n-TBI groups found to be significantly different from healthy controls (*P* < 0.05, Bonferroni corrected). Figure 4c details the P300 latency of each patient with severe brain injury (TBI or n-TBI) or stroke. Participant 8, who was partially responsive, did not demonstrate a P300 response despite an intact N100 and some behavioural signs of hearing (e.g. startle response to out of sight noise). This demonstrates an important proviso for the HCS and ERPs in general: while positive HCS results have the potential to provide informative data—a negative result such as obtained from Participant 8 (clearly partially responsive) must be treated as an unknown rather than as an indication of absence of awareness. The box plots in Fig. 4d show the distribution of P300 latencies in each group, including healthy (*N* = 100), fully conscious (*N* = 5), partially conscious (*N* = 10), non-responsive (*N* = 3) and comatose (*N* = 1) with individual data points specified.

**Figure 5.**
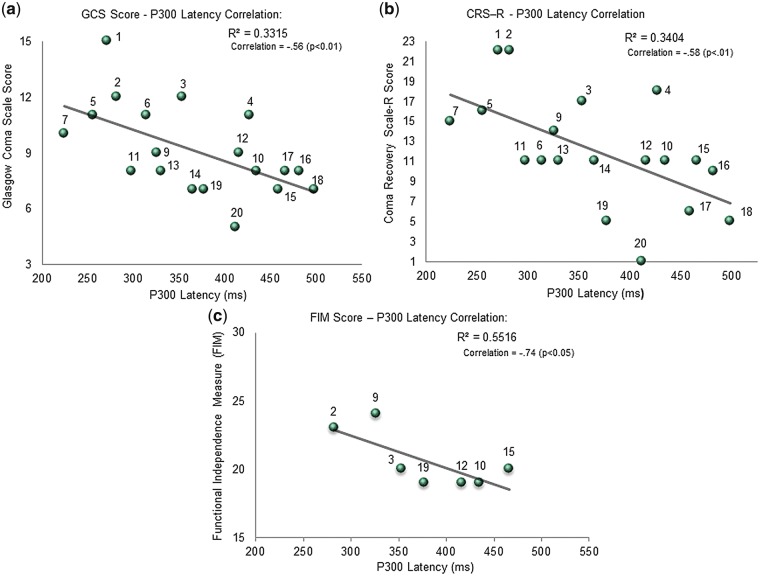
(**a**) P300 latencies in ms (x-axis) significantly (negatively) correlated with Glasgow Coma Scale (GCS) (y-axis) on 2-tailed tests with participant numbers labelled for 19 patients. (**b**) P300 latency in ms (x-axis) significantly (negatively) correlated with Coma Recovery Scale-Revised (CRSR) (y-axis) on 2-tailed tests with participant numbers labelled for 19 patients. (**c**) P300 latencies in ms (x-axis) compared with Functional Independence Measure (FIM) score (y-axis) with participant numbers labelled for 7 patients. Although significance was not reached on the 2-tailed test, the results were significant on the 1-tailed test (*r* = −0.74, *P* < 0.05).

**Figure 6.**
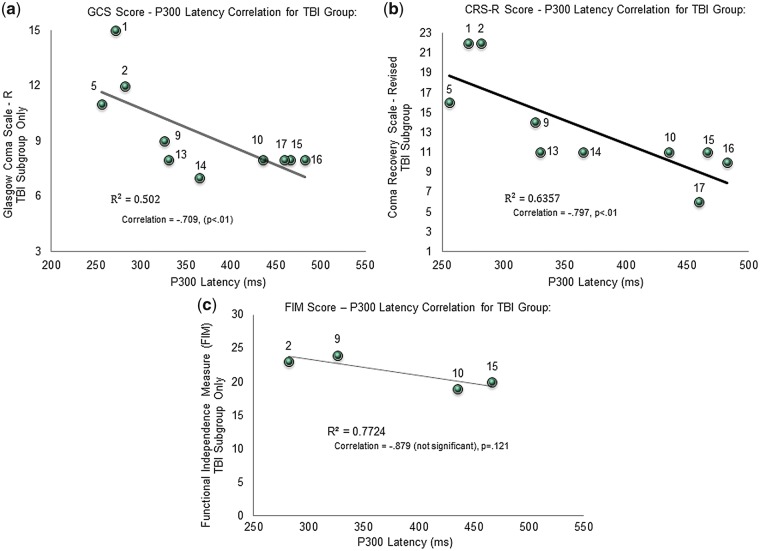
(**a**) Demonstrates a significant negative correlation between the P300 latency in ms (x-axis) and Glasgow Coma Scale Score (y-axis) for 10 TBI participants (numbered in figure). (**b**) Demonstrates a significant negative correlation between the P300 latency in ms (x-axis) and Coma Recovery Scale-Revised Score (y-axis) for 10 TBI participants (numbered in figure). (**c**) Demonstrates a negative correlation between P300 latency (x-axis) and the Functional Independence Measure (FIM) (y-axis) for the 4 TBI participants (numbered in figure) who were scored with this clinical measure but the correlation is not significant.

Two-tailed bivariate Pearson correlations demonstrated that the P300 latency correlated significantly with the GCS (*n* = 19, *r* = −0.56, *P* < 0.01), CRS-R Total Score (*n* = 19, *r* = −0.58, *P* < 0.01) and FIM score (*n* = 7, *r* = −0.74, *P* < 0.05, 1-tail). See Fig. 5a–c. When only the TBI sub-group was considered, the P300 latency continued to correlate negatively with all clinical scores (*P* < 0.01) on 2-tailed tests except for the FIM (*r* = −0.88), which did not reach significance. However, given FIM scores were only available for 4 of the TBI participants, this result is likely compromised by reduced statistical power. See Fig. 6a–c.

## Discussion

The primary objectives of this study were to examine the feasibility of using the HCS for POC evaluation in sub-acute settings nationwide in Canada. The findings demonstrated that HCS testing at POC is feasible in sub-acute settings, with patient P300 latencies significantly delayed relative to normative health control P300 latencies (Hypothesis 1). As expected, significant correlations were established across clinical measures (Hypothesis 2). Importantly, further correlational analyses showed significant linear relationships between individual patient P300 latencies and established clinical measures (Hypothesis 3). Bootstrapping confidence intervals for P300 latencies were calculated within both healthy and patient samples, and confidence intervals did not overlap between the healthy and patient samples. This result confirmed that it is possible to obtain objective brain measures in front-line, subacute POC assessment and monitoring applications—even in patients with severe movement impairments. However, HCS utility and diagnostic accuracy in early diagnosis remain unknown at this time.

In an important study, Chennu *et al.* (2013) demonstrated the P300 marker of attention (exogenous and/or endogenous) in some behaviourally unresponsive patients. The authors evaluated P3a and P3b in 30 patients and 8 healthy volunteers. Nine subjects were rejected due to heavy artefact noise. In the 21 remaining patients, the authors showed evidence of exogenous and endogenous attention in a patient in an unresponsive wakeful state and exogenous attention in three patients in minimally conscious states. The unresponsive patient and two of the three minimally conscious patients subsequently demonstrated command following during tennis imagery tasks on fMRI. Whereas the focus of the aforementioned study was to determine the presence or absence of the P300 family of responses in the specified time window, this HCS study evaluated the correlation between behavioural scales and P300 latency. Like in the auditory HCS study, Chennu *et al.* (2013) reported a high level of inconsistency in the responses across the patient group. Kouchoubey and Pavlov (2018) completed a systematic review and meta-analysis of the relationship between brain data and outcome in DoC including 47 publications. Surprisingly, their results demonstrated that P300 and fMRI showed poor prognostic effects. This however, does not negate the importance of the measures in understanding the nature of a patient’s condition. While it is clear that P300 latency allows us to make assumptions about information processing, other ERP measures such as N400, may be more useful for prognostication. Related studies by our group (Hajra *et al.* 2018; Pawlowski 2018) and others (Steppacher *et al.* 2013) explored the potential of using N400 as a physiological indicator of masked conscious awareness with promising results.

Clinical settings have not capitalized on the potential of electrophysiology to contribute to the process of DoC evaluation, status monitoring and care planning/service designation. There is a pressing need for an easily deployed, low-cost, non-invasive and repeatable objective assessment strategy that can be used to serially monitor conscious awareness at the single patient level. Given that a patient’s state of conscious awareness hinges on many factors and can change over time; it makes sense to assess and monitor these patients once they are medically stable in the sub-acute phase. In recent years, several measures for capturing task related neural activity such as alpha band power, spectral edge frequency and mean spectral frequency have been identified (Bai *et al.* 2017; Song *et al.* 2018). Additional measures have also been reported for evaluating blink related oscillation effects, with some demonstrated efficacy in differentiating between vegetative and minimally conscious state patients (Bonfiglio *et al.* 2013, 2014; Liu *et al.* 2017). As an important first step, André-Obadia *et al.* (2018) have proposed recommendations for EEG and evoked potentials in comatose patients. Future work will also explore spectral markers derived from HCS data to further characterize the rich data available and this may yield complementary information. It may also be useful to explore the utility of the device in acute settings, although in acute settings many factors (consciousness-altering drugs, coexisting medical problems, etc.) can reduce the feasibility of the test and compromise the validity of the results.

The HCS uses a portable EEG system to rapidly deliver a compressed ERP sequence at POC without interrupting daily clinical routines or exhausting the patient. Given that this system does not rely on overt responses, it can be done independent of behavioural responses and therefore is not confounded by ‘motor-mind disconnection’. It is imperative that patients in minimally conscious or locked-in states receive the stimulation and rehabilitation necessary to maximize their odds of improvement (Giacino 2004; Fins *et al.* 2016; Illman and Crawford 2017).

The current study has a number of caveats. Given the relative rarity of disorders of conscious awareness and challenges inherent to fluctuating health status, securing an adequate sample size was challenging. This is especially the case when the objective is to evaluate a deployable HCS across a wide array of settings. Nonetheless, similar studies have employed a wide range of sample sizes ranging from 8 (Bekinschtein *et al.* 2009) to 173 (Sitt *et al.* 2014). Several studies targeting the P300 family of responses (Cavinato *et al.* 2011; Chennu *et al.* 2013; Ragazzoni *et al.* 2013) have used sample sizes similar to the present study.

Another caveat relates to selection bias. In this study, patients were referred by caregivers or therapists who likely preferentially referred patients suspected to have some degree of awareness. Given this bias, separate validation studies would be required for patients with absolutely no clinical or behavioural indicators of consciousness. Further work is required to model effects across different centres to better understand key influencing factors (e.g. hardware, environment, data collection protocols, etc.).

Due to the nature of conscious awareness, studies of DoC also face inherent challenges of sensitivity and specificity. Diagnosis occurs at the individual patient level so although showing group differences is necessary for a tool to be diagnostically useful, it is not sufficient. However, because a ‘gold standard’ for the assessment of consciousness independent of behaviour does not exist, it is very difficult to validate a new tool. Further, as is the case with other technologies that have been trialled for diagnosing conscious awareness such as fMRI, the HCS is sensitive to specific markers like the P300, but lacks specificity in the event of negative results. Therefore, negative test results must be considered inconclusive. Even though ERPs can eliminate reliance on overt responses, participation requires basic attention, sensory, perceptual, and often, receptive language capacity. A breakdown can occur at the input stage, even if internal awareness exists. In order to mitigate this confound, a larger, multi-sensory diagnostic battery is necessary.

## Supplementary Material

Supplementary Data
