# Supplementary figures and images for: Point-of-care brain injury evaluation of conscious awareness: wide scale deployment of portable HCS EEG evaluation
Source: Neurosci Conscious. 2018 Nov 23;2018(1):niy011. doi: 10.1093/nc/niy011 (PMC6251986; doi:10.1093/nc/niy011)

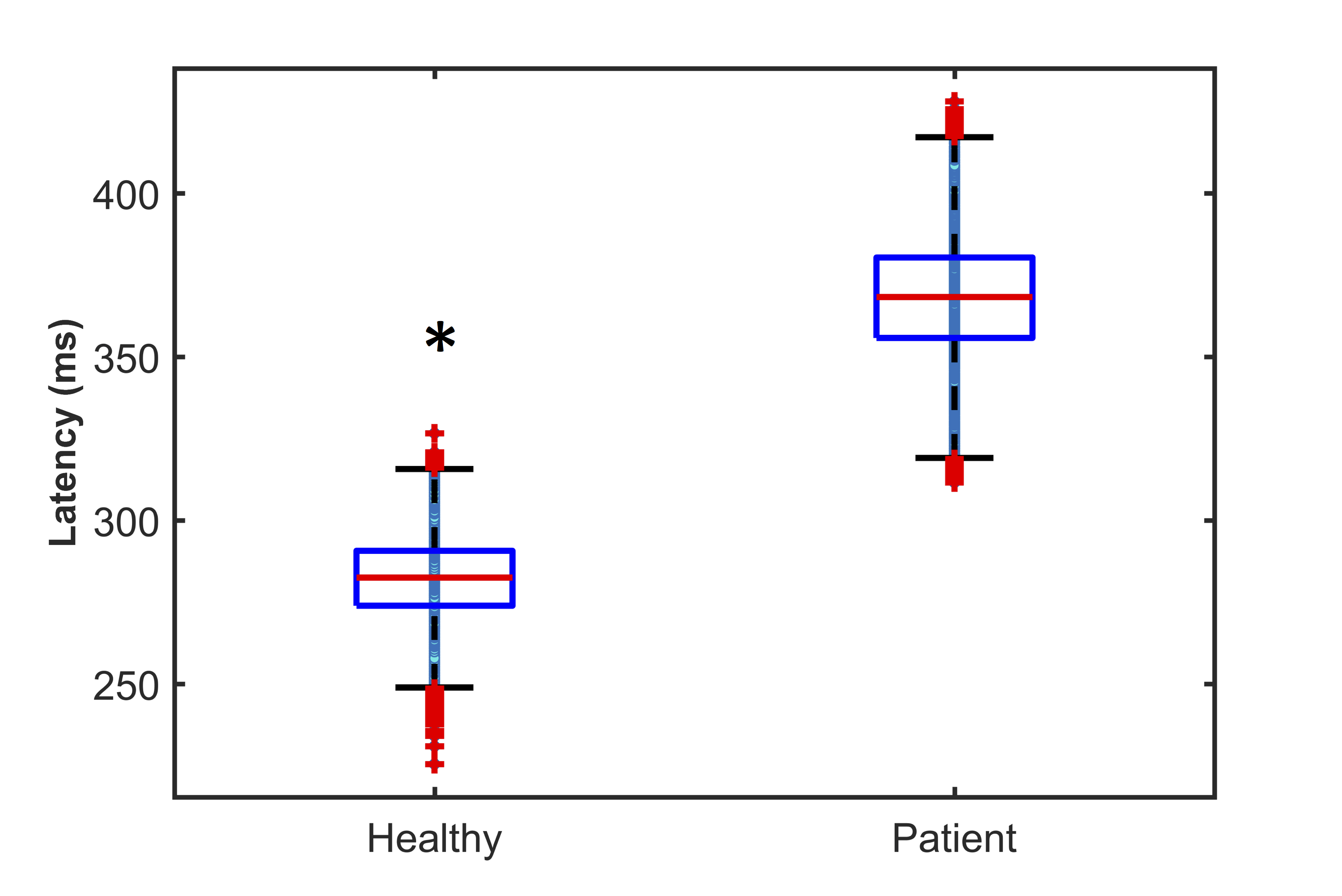

Supplement: Supplementary Data [file niy011_figure_s1.png]
